# Use of Leukocyte Platelet (L-PRF) Rich Fibrin in Diabetic Foot Ulcer with Osteomyelitis (Three Clinical Cases Report)

**DOI:** 10.3390/diseases6020030

**Published:** 2018-04-24

**Authors:** Alessandro Crisci, Giuseppe Marotta, Anna Licito, Edda Serra, Giulio Benincasa, Michela Crisci

**Affiliations:** 1 Unit of Dermosurgery Cutaneous Transplantations and Hard-to-Heal Wound, “Villa Fiorita” Private Hospital, 81031 Aversa, CE, Italy; 2 School of Medicine, University of Salerno Italy, 84084 Fisciano, SA, Italy; 3 Institute for the Studies and Care of Diabetics, Abetaia, 81020 Casagiove, CE, Italy; infodue@diabetologia.it (G.M.); arianna.licito@hotmail.it (A.L.); eddaserranet@libero.it (E.S.); 4 Pathological Anatomy, “Pineta Grande” Private Hospital, 81030 Castelvolturno, CE, Italy; giulio.benincasa@pinetagrande.it; 5 Faculty of Medicine and Surgery, Vasile Goldis Western University of Arad, 310025 Arad, Romania; criscimichela96@gmail.com

**Keywords:** osteomyelitis, buffy coat, growth factor level, platelet-rich fibrin, thrombocyte concentrate

## Abstract

In this study, the use of fibrin rich in leukocytes and platelets (L-PRF) was explored to heal osteomyelitis ulcers in a diabetic foot. The goal was to standardize the utilization of L-PRF in patients with osteomyelitis to direct it for healing. L-PRF was obtained autologously from the peripheral blood of the diabetic patients (*n* = 3) having osteomyelitis and skin lesions for at least six months. The L-PRF and supernatant serum were inserted into the skin lesion to the bone after a surgical debridement. The evolution of lesions over time was analyzed. All three patients showed positivity to the Probe-to-Bone test and Nuclear Magnetic Resonance detected cortico-periosteal thickening and/or outbreaks of spongy cortical osteolysis in adjacency of the ulcer. The infections were caused by Cocci Gram-positive bacteria, such as S. Aureus, S. β-hemolytic, S. Viridans and Bacilli; and Gram-negative such as Pseudomonas, Proteus, Enterobacter; and yeast, Candida. The blood count did not show any significant alterations. To date, all three patients have healed skin lesions (in a patient for about two years) with no evidence of infection. These preliminary results showed that L-PRF membranes could be a new method of therapy in such problematic diseases. Overall, the L-PRF treatment in osteomyelitis of a diabetic foot seems to be easy and cost-effective by regenerative therapy of chronic skin lesions. In addition, it will improve our understanding of wound healing.

## 1. Introduction

Osteomyelitis (OM) (bone infection) refers specifically to a bone marrow infection in contrast to osteitis wherein the periosteum or cortical surface becomes infected through a penetrating wound or ulcer. Despite these differences, both are clinically diagnosed or treated in a very similar manner. There have been many studies on the diagnosis of OM and, most importantly, on the complication of diabetic foot ulcers (DFU). OM is a comorbidity of the DFU, which almost always arises from a contiguous wound or an ulcer of the foot [1].

Bone and joint infections are frustrating and exasperating for both patients and doctors. A desirable success rate in antimicrobial therapy in most infectious diseases has not yet been achieved. The different types of osteomyelitis require diverse surgical and medical therapeutic approaches. These types include, in order of decreasing occurrence, osteomyelitis secondary to the adjoining point of infection (after insertion of a joint prosthesis or trauma, surgery, etc.), which is secondary to vascular insufficiency (in diabetic foot infections), that of hematogenous derivation. Chronic osteomyelitis is related to the vascular necrosis of bone and the formation of sequestrum (dead bone), and surgical debridement is necessary for medication in addition to antibiotic therapy. In contrast, acute osteomyelitis could respond well to antibiotics alone. Commonly, a multidisciplinary approach is required for success, involving expertise in infectious diseases, orthopedic surgery, and plastic surgery, as well as vascular surgery, particularly in complex cases with soft-tissue loss [2,3].

The platelet-rich fibrin (PRF), described by Choukroun [4], is a new therapeutic approach using platelet concentrates, with a simplified design and requiring minor artificial biochemical changes. This technique does not require anticoagulants, thrombin, or any other gelling agent, which renders it different from natural blood, centrifuged without additives [5]. Although platelet and leukocyte cytokines play an important role in the biology of this biomaterial, the supporting fibrin matrix certainly constitutes the decisive factor of the real therapeutic potential of L-PRFs. Within a few minutes, the absence of an anticoagulant allows the activation of most of the platelets contained in the sample to trigger the coagulation cascade.

The use of L-PRF in the DFU with OM has not yet been attempted, thus, this article describes the first report of this approach.

## 2. Materials and Methods

### 2.1. Patients

L-PRF membranes were obtained from the peripheral blood of three patients (all diabetic) having osteomyelitis with skin lesions existing for at least six months. Therefore, the evolution of lesions over time toward healing was analyzed. All treated patients gave their informed consent for inclusion before participating in the study.

**Figure 1:**
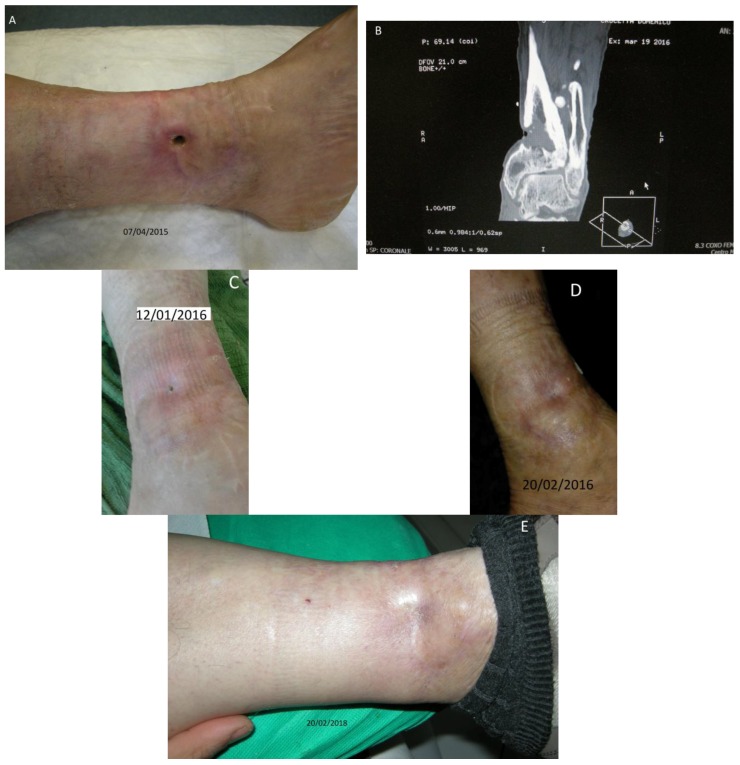
Case No. 1. (**A**,**C**,**D**,**E**) Different moments of the wound healing, stable after two years; (**B**) NMR of the patient with the bone lesion.

Patient No. 1: C.D., 68-year old male, diabetic for 25 years; Body Mass Index (BMI) = 35.5; non-smoker; nonalcoholic; having a cutaneous ulcer on the inner side of the left leg for more than 20 years (Figure 1A–E), penetrating the structure of the tibia, with bone lesion (Figure 1B). The patient was currently taking glicazide 60 mg at breakfast, sitagliptin phosphate monohydrate/metformin hydrochloride 50 mg + 1000 mg at lunch and dinner; did not present sensory-motor polyneuropathy; fasting blood glucose ~150 mg/dL; Urea 29 mg % (normal range nr: 10–50); HbA1c DCCT = 6.7% (nr: 4–6); HbA1c IFCC = 50 mmol/mol (nr: 10–50).

**Figure 2:**
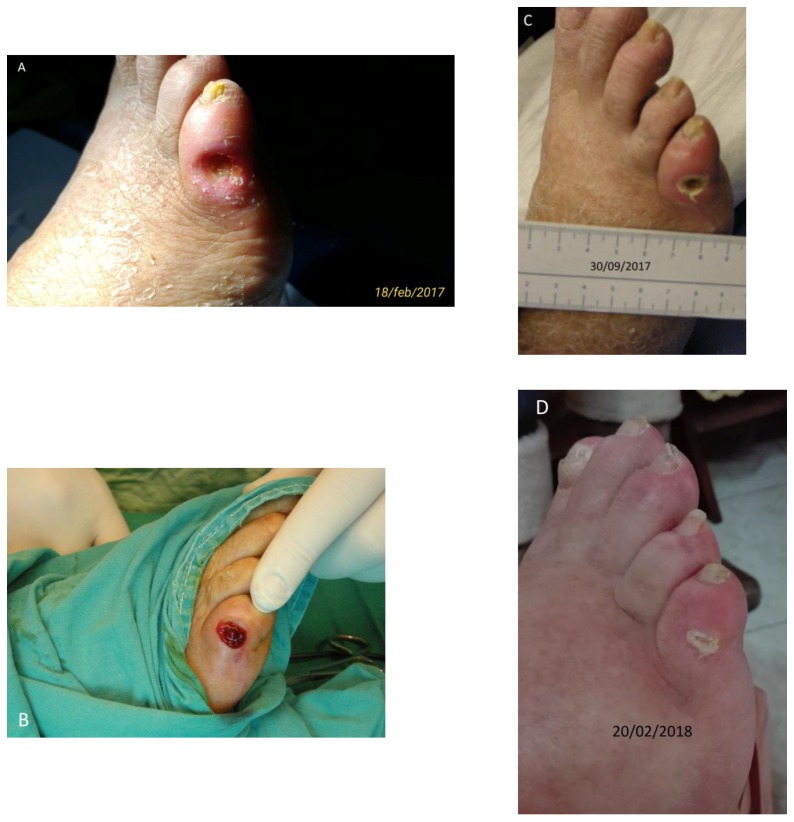
Case No. 2. (**A**,**C**,**D**) Different moments of the wound healing, stable after one year; (**B**) The lesion after L-PRF grafting.

Patient No. 2: D.F.F., a 71-year-old female, diabetic for last 40 years; BMI = 39; former smoker; moderately alcoholic; since the age of 33 years with Arterial Hypertension; from the age of 46 years she was reported to have Hypertensive Cardiopathy with atrial fibrillation and flutter; of the leg as recorded by Ecocolordoppler assay “Obstructive arterial disease”; by Angio-CT “Femoral Common and Superficial right stenotic, tibioperoneal axis dx and sn not highlighted”; bearing a cutaneous ulcer at the 5th toe of right foot for about one year penetrating the bone structure of the proximal phalanx (Figure 2A–D). The patient was currently taking acetylsalicylic acid 100 mg/day, rapid insulin 3 mL 100 U/M (15 U at breakfast, 25 U at lunch, 20 U at dinner); slow insulin 3 mL 100 IU/M (40 U in the evening); also presenting severe sensorimotor polyneuropathy (class III); fasting blood glucose ~300 mg/dL; Urea 58 mg% (nr: 10–50); HbA1c DCCT = 8.4% (nr: 4–6); HbA1c IFCC = 68 mmol/mol (nr: 10–50).

**Figure 3:**
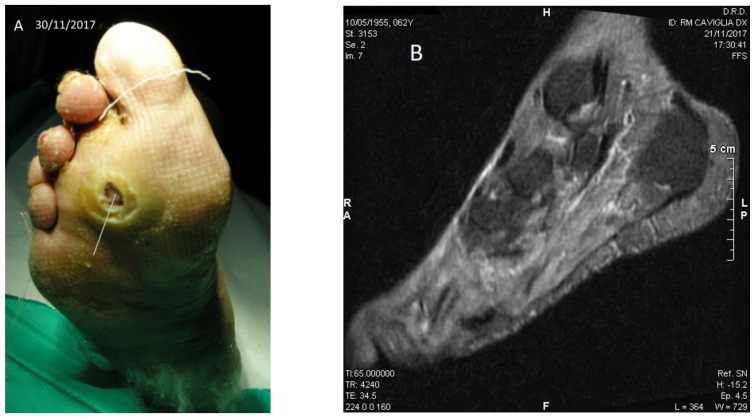

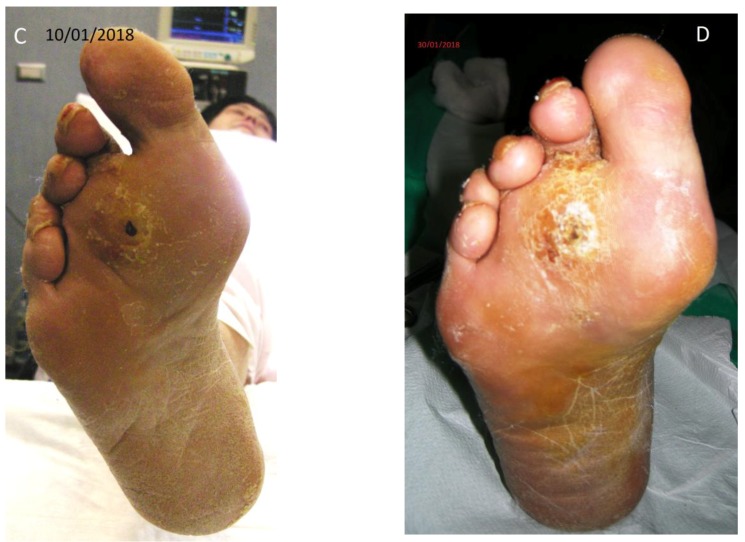
Case No. 3. (**A**,**C**,**D**) Different moments of the wound healing, stable after six months; (**B**) NMR with the bone lesion at the third metatarsal ray.

Patient No. 3: P.C., 63-year-old female; diabetic since age 39; BMI = 36.3; former smoker; non alcoholic; from the age of 51 years Chronic Renal Failure; from the age of 60 she presented atrial fibrillation and flutter; carrying a plantar ulcer on the right foot for about three years (Figure 3A–D), penetrating the bone structure of the third distal metatarsal ray (Figure 3B). The patient was currently taking acetylsalicylic acid 100 mg/day, rapid insulin 3 mL 100 U/M (3 U at breakfast, 6 U at lunch, 8 U at dinner); slow insulin 3 mL 100 IU/M (14 U in the evening); also presented slight sensorial-motor polyneuropathy (class I); fasting blood glucose ~57 mg/dL (recurrent hypoglycemia); Urea 112 mg% (nr: 10–50); HbA1c DCCT = 7.9% (nr: 4–6); HbA1c IFCC = 63 mmol/mol (nr: 10–50).

### 2.2. L-PRF Preparation

The blood was collected without anticoagulant or a gel separator in plastic-coated glass test tubes (BD Vacutainer tubes for serum 9.0 mL, Milan, Italy), for the production of L-PRF clots and membranes. The blood was collected quickly with a needle to Vacutainer tubes (22″ average value, of less than 25″ per tube) and immediately (within 1 min) centrifuged at a temperature greater than 21 °C (between 21 and 30 °C) according to the protocol described earlier [6,7,8,9,10]. Using an L-PRF Wound Box, the compression process of the membrane in the clots was performed through a slow and homogeneous slight compression, and the final membrane was maintained homogeneously wet and soaked in serum.

The technique to obtain L-PRF is very simple and requires only a homogeneous blood sample and a table-top centrifuge [6,7,8,9,10]. The protocol is as follows: the blood samples are collected in 9 mL tubes without anticoagulant or gel separator, and are immediately centrifuged according to the following program: 30 s acceleration, 2 min at 2700 rpm, 4 min at 2400 rpm, 3 min at 3000 rpm, and 36 s deceleration and stopping.

After the centrifugation, three layers were formed in the tube: the red blood cells at the bottom, a fibrin clot that represents the PRF in the middle, and the acellular plasma at the top. The fibrin clot was extracted from the tube with sterile forceps and the PRF was obtained by removing the red clot from its lower end. The success of this technique depends on the blood collection and the transfer speed in the centrifuge [6,7,8,9,10].

This mild method offers an efficient extraction avoiding much loss of the growth factors. The PRF Wound Box is available on the market in a variety of shapes and exerting pressure. Through a compression plate, different pressures according to the weight can be obtained giving rise to a membrane of varying thickness, width, and length. The L-PRF Wound Box [6,7,8,9,10] designed by us is made of a metal container 17.5 × 7.6 × 2 cm, containing a perforated steel plate of 150 × 68 × 1.5 mm. There is a second steel plate, which acts as a compressor, 150 × 68 × 1.5 mm, having a weight of 148 grams. The second shaped plate exerts a pressure of 142.437 Pa/cm^2^. In this study, the compression was exerted on the clot for 2 min to produce membranes. Each membrane was divided into three equal-sized areas: proximal (head), center (body), and distal (tail).

Only the proximal part of the membrane was used [9,10].

### 2.3. Analysis

A blood sample was also taken from each patient to perform a blood count using K_3_EDTA 5.4 mg tubes (VacuMed, BD, Milan, Italy).

In accordance with the study by Peck et al. [11], three blood samples were taken from the left brachial vein of each patient through an 18-gauge needle, one for the blood count and two for the production of PRF. The analyses were performed with a Cell Dyn 3500 R cell counter (ABBOTT, Milan, Italy).

The diagnostic assessment of OM in the patients was performed first of by the Probe-to-Bone method (PTB) and then through an MRI (see figures below) and a bone cell culture.

### 2.4. L-PRF Grafting Procedure

Each of the three patients, in the operating room, underwent surgical debridement with the removal of non-viable tissues and possible bone fragments at the bottom of the lesion. The whole procedure was carried out under subarachnoid anesthesia. The surgical prevention procedure included an appropriate suspension of anticoagulant drugs for at least seven days and substitution with low molecular weight heparin subcutaneously. Further, the ulcer specimens were harvested for micro-bacterial analysis. No peripheral vasodilatory drugs were used.

Surgical areas were disinfected with a 50% mixture of hydrogen peroxide/iodopovidone and appropriate hemostasis controls with thermocautery were considered. The ulcers were medicated by applying L-PRF prepared in the form of membranes after coagulation compression for 2 min. The supernatant derived from the squeezing was collected by the L-PRF Wound Box with a sterile 10 cc syringe and was inserted into the skin lesion up to the figures carefully together with the proximal III of the L-PRF membrane (Figure 2B) [9,10].

The dressing was done by using fat gauze, sterile gauze, germanic cotton and adhesive elastic bandage. Post-surgical treatment included levofloxacin 500 mg cp, 1 cp per day for five days and low molecular weight heparin (enoxaparin sodium) for seven days. In addition, based on the results of the culture and the antibiogram, specific antibiotics were added for general use for 15 days.

The first dressing was performed after seven days.

For two patients (no. 1 and no. 2), it was necessary to re-perform the procedure after 40 days.

## 3. Results

All three patients had positive Probe-to-Bone tests. The Nuclear Magnetic Resonance (NMR) detected cortico-periosteal thickening and/or focal points of cortico-cancellous osteolysis with reduced signal intensity in adjacency of cutaneous ulcer. Further, edema due to the septic inflammation and abscess of soft tissues was also observed.

Several pathogens have been found in OM and bacteria are the most frequent. In rare cases, fungi, protozoa, and viruses could also be present.

In our patients, multiple pathogens were detected at the same time. Gram-positive bacteria were found in 52% of cases. It included *S. aureus* (15.6%), beta-hemolytic *Streptococcus* (12.1%), *S. viridans* (7.1%) and *Bacilli.* Gram-negative bacteria included *Pseudomonas* (10.6%), *Proteus* (7.8%), and *Enterobacter* (5.7%). *Candida* was present in 2.8% cases.

The hemochromocytometric examination did not show any considerable changes.

To date, all three patients have healed skin lesions (with follow up of two-year) and there is no sign of infection and relapses.

## 4. Discussion

During the L-PRF preparation, blood clotting begins instantly upon coming in contact with a glass surface due to the lack of anticoagulant. If the time required to collect blood and process for centrifugation is prolonged, the fibrin polymerization becomes widespread in the tube and only a small part of the blood clot could be obtained without consistency (PRF-like). Therefore, the blood collection must be followed by immediate centrifugation, which is essential for good PRF yield. It should be formulated to obtain a homogeneously-moisturized thick membrane and an exudate rich in platelets, leukocytes, vitronectin, and fibronectin expressed by fibrin clots [12,13,14].

At first, fibrinogen is concentrated in the upper part of the tube, until by circulating autologous thrombin, it is transformed into a network of fibrin. The result is a fibrin clot containing the platelets located at the center of the vial, between the lower layer of red blood cells and the upper one of the low cell-containing plasma. The L-PRF clot is then placed on the grill in the L-PRF Box and covered with the compressor cover. This produces a membrane of autologous fibrin in approximately one minute. The L-PRF Box is designed to give a constant thickness of membranes that could remain hydrated for many hours and allow the recovery of the serum exudate expressed by fibrin clots, which are rich in proteins, such as fibronectin (Fn) and vitronectin (Vn) [5]. The L-PRF clot is produced by a natural polymerization process during centrifugation, and its natural fibrin architecture seems to be responsible for a slow release of growth factors and glycoproteins from the matrix (≥7 dd). The adhesive proteins fibrinogen (Fg), Fn, Vn, and thrombospondin–1 (TSP–1) are abundant on the fibrin structure. Among the growth factors stored in platelets, which are essential for the repair of wounds, are PDGF, with -AB and -C. There are also present VEGF-A, TGF-β1, FGF–2; EGF, HGF, and insulin-like growth factor–1 (IGF–1) [15,16].

By analyzing three proinflammatory cytokines (IL-1β, IL-16, TNF-α), an inflammatory cytokine (IL-4) and angiogenesis promoter (VEGF), it has been shown that PRF could be a crux in immune modulation with the possibility of inflammation control, considering the genetic predisposition [17,18]. Overall, the L-PRF is mechanically resistant, able to support loads, has a capacity of two times to be stretched under tension and retains surgical sutures well enough (it deforms significantly before the laceration) [19].

Finally, we believe that the L-PRF-based treatment of OM in the diabetic foot ulcer will progress our understanding of wound healing, particularly in the regenerative therapy of chronic skin lesions. The results obtained from our patients show that L-PRF membranes can be a new method of therapy in this difficult treatment pathology.

As the number of patients treated with this method is very low, we intend to perform a randomized trial to confirm the clinical effect of L-PRF in OM of DFU. In addition, we will also test the antibacterial proprieties of the L-PRF and would like to perform an annual follow-up to monitor the evolution of lesions in these three patients, even with the use of Nuclear Magnetic Resonance.
